# Identification of Key Components Responsible for the Aromatic Quality of Jinmudan Black Tea by Means of Molecular Sensory Science

**DOI:** 10.3390/foods12091794

**Published:** 2023-04-26

**Authors:** Qingyang Wu, Ziwei Zhou, Yining Zhang, Huiqing Huang, Xiaoxi Ou, Yun Sun

**Affiliations:** 1 Key Laboratory of Tea Science in Fujian Province, College of Horticulture, Fujian Agriculture and Forestry University, Fuzhou 350002, China; doris1831036881@126.com (Q.W.);; 2 College of Life Science, Ningde Normal University, Ningde 352000, China; zwchow92@126.com

**Keywords:** Jinmudan black tea, aroma quality, PLS, GC-O

## Abstract

A fruity aroma is regarded as an important factor in the evaluation of black tea quality. However, the compounds contributing to a particularly fruity aroma still garner less attention. In this study, we aimed to identify the aroma-active compounds of the peach-like aroma of Jinmudan black tea (JBT). We used gas chromatography–mass spectrometry (GC-MS) to reveal the profile of the chemical compounds integrated into JBT and identified terpenoids, heterocyclic, and esters that contribute to its floral and fruity aroma. Under the PCA and PLS-DA modes, JBT and Fuyun NO. 6 black tea (FBT) can be divided into two classes, respectively (class 1 and class 2); several compounds, including indole, methyl salicylate, and δ-decalactone, have a higher VIP value (Variable Importance in Projection), and it has been found that δ-decalactone was the characteristic aromatic compound of peach fruit. Gas chromatography–olfactometry (GC-O) and the odor activity value (OAV) indicated that, in JBT, linalool, phenylacetaldehyde, and δ-decalactone could be considered aroma-active compounds (AACs). However, in FBT, the high content of heterocyclic compounds contribute to its caramel-like aroma. As for the biochemical compounds measurement, JBT has a higher content of theaflavins (TFs), thearubigins (TRs), and flavonoids. These results provide a theoretical basis for the quality and processing improvement in JBT.

## 1. Introduction

After water, tea is the most-consumed beverage worldwide. The quality of tea is regulated by several known factors, from the pre-harvesting to post-harvesting stages, such as the cultivar [1], natural environment [2], harvesting seasons [3], biology stressors [4], processing methods [5,6,7], and the storage environment [8], among others.

Black tea is a type of fully fermented tea that is consumed globally due to its unique taste and aroma [9]. The usual process of black tea production consists of four stages: withering, rolling, fermentation, and drying. Certain studies have focused on the quality of traditional black tea (Congou black tea), and the formation mechanism of flavor has been further investigated. Studies have focused on the influence of different cultivars, seasons, and production areas of traditional black tea [10]. The mode of quality measurement has also been constructed based on the key characterization index of black tea samples at withering and fermenting stages [11,12]. Molecular sensory approaches have also been widely applied in the identification of flavor compounds in traditional black tea. Furthermore, E-nose combined with GC×GC-TOF MS (Comprehensive Two-dimensional Gas Chromatography/time-of-flight Mass Spectrometry) were applied in the identification of characteristic aroma compounds at the drying stage [13]. SPEM-GC-MS (Head Space-Solid Phase Microextraction–Gas Chromatography Mass Spectrometry) combined with chemometrics were also investigated regarding the influence of various water temperatures on the aroma at the brewing stage [14]. An E-tongue was also used in the classification of traditional black tea [15]. The mechanisms of the infusion of sensory flavor in tea have also been investigated, and it has been found that the accumulation of caffeine, γ-aminobutyric acid, rutin, and other compounds, may forbid sweet and mellow flavors [16].

**Figure 1:**
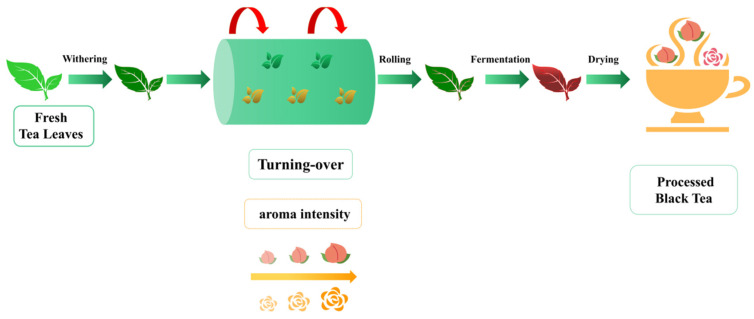
The processing methods of innovative black tea. Legends and arrows represent the change in the conditions and color of tea leaves along with the manufacturing process. The green cylinder-shaped symbol represents the manufacturing machine with half-full tea leaves; the orange arrow represents that during the turning-over period of innovative black tea, the floral and fruity aroma intensity became increasingly strong. The orange cup-shaped symbol represents the processed, innovative Jinmudan black tea with a highly floral and fruity (peach-like) aroma.

Different to traditional black tea, the processing methods of innovative black tea additionally include turning over after the withering period (Figure 1), a method initially originating from the processes used for oolong tea. It was found that during the turning-over period, the aromatic volatile compounds formatted and accumulated, finally contributing to the floral and fruity aroma of the processed tea [17,18]. Moreover, innovative black tea tends to be processed with aroma-enriched tea cultivars, such as *Camellia sinesis* cv. Jinmudan, Jinguanyin, Huangguanyin, and other specific cultivars. Among them, *Camellia sinesis* cv. Jinmudan (JMD) is a hybrid tea cultivar, and its parents are Tieguanyin and Huangdan. Compared to other previously planted tea cultivars, such as Fuyun No.6, Iinmudanm has gained a reputation for its resistance and suitability for various tea categories. Consequently, Jinmudan has been used to process high-quality oolong tea, white tea, and definitely black tea [19]. Briefly, the innovative black tea process used for JMD produces a mellow and fresh taste as well as a floral and fruity fragrance, which provides the characteristics of a peach-like aroma in particular samples.

However, most studies have only characterized the profiles of tea compounds rather than the aroma-active compounds. In addition, the aromatic characteristics and odor thresholds of certain components were not considered as conditions that could actually contribute to the tea’s aroma. Therefore, our goal was to focus on defining the aroma quality of Jinmudan black tea and to identify the components responsible for its characteristic floral and fruity aroma. In addition, we aimed to characterize the aroma-active compounds of the peach-like aroma at a molecular sensory level.

In order to achieve our aims, the GC-O (gas chromatography–olfactometry) technique was applied. The GC-O technique is an analytical approach that combines olfactory and instrumental detection that allows the simultaneous acquisition of both the chemical compounds and the odor characteristic information of samples. Due to the combination of precision detection and sensitivity of human olfaction, this technique has been widely applied in fields as diverse as the food industry [20,21], cosmetics industry [22], and chemical industry [23,24], among others. In recent years, it has also been gradually applied to tea aroma evaluation [25,26]. Thus, GC-O represents a creditable and innovative approach to identifying specific aromatic compounds in tea.

Here, we characterized innovative JBT using a molecular sensory approach. Sensory evaluation and the biochemical compounds of JBT were investigated to provide a fundamental overview about the flavor of JBT. Furthermore, the aroma profile of JBT was described by means of GC-MS detection combined with PCA and PLS-DA analysis. The results obtained via GC-O technology contributed to the identification of the key aroma-active compounds responsible for the peach-like aroma quality of JBT, and the odor activity values (OAV) and aroma-active impact (ACI) were also calculated. Our study thus provides a basis of knowledge for further investigations of the aroma precursors and dynamic changes in volatile compounds during the tea processing period. It also serves as a feasible reference for the spread and cultivation of Jinmudan tea plants as well as the oriented manufacturing of Jinmudan black tea.

## 2. Material and Methods

### 2.1. Tea Samples

A total of ten kinds of black tea samples were collected in Fu’an City, Fujian, China, in 2022. All of the tea samples were processed by different tea factories, but all of them were processed from *Camellia sisnensis* cv. Jinmudan and were listed from JBT 1 to JBT 10. Additionally, these ten kinds of JBT were produced using innovative methods, which included withering, turning-over, rolling, fermentation, and drying.

Three kinds of black tea made from *Camellia sisnensis* cv. Fuyun No.6, processed by different factories, were used as a control, listed as FBT 1 to FBT 3. Different from JBT, these three FBTs were produced using traditional methods, which only included withering, rolling, fermentation, and drying, except for the turning-over method.

All of the samples were stored in a refrigerator at 4 °C until further analysis.

### 2.2. Sensory Evaluation

The sensory evaluation of black tea samples was conducted by five tea tasters, including one male and four females, who were trained by professional organizations and all of whom passed the tea evaluation examination. The sensory evaluations were performed in a clean and dry room at 26 °C. As for the tea preparation, 3.0 g of the processed black tea samples was immersed in 150 mL of boiled water. After 5 min, the tea infusion was transferred into the tea bowl. During the immersing period, the quality of the black tea samples, especially the aroma characteristics, was described and recorded in detail.

### 2.3. Analysis of Color Difference of the Tea Infusions

The color parameters were conducted with a colorimeter (NR200, Sanenshi Technology Co., Ltd., Shenzhen, China), the samples were scanned three times, and finally, the mean was given. The color was quantified according to the Hunter color values L*, a*, b*, and L* represents the change of lightness from black (0) to white (100) as well as a* and b*, respectively, represent red (+a*) and green (−a*), yellow (+b*), and green (−b*).

### 2.4. Biochemical Compositions Quantification

The detection of biochemical compounds in both JBT and FBT, including polyphenols, free amino acids, caffeine, theaflavins (TFs), thearubigins (TRs), theabrowings (TBs), flavonoids, and water-extractable substances, was conveyed based on various National Standards of the Republic of China (Table 1).

### 2.5. Gas Chromatography Mass Spectrometry (GC-MS) Analysis

#### 2.5.1. Sample Preparation and Aroma Extraction

The materials were harvested, weighed, immediately frozen in liquid nitrogen, and stored at −80 °C until needed. Before the milling procedure, they were first immersed in dry grinding cans in liquid nitrogen for 1~2 min until there were no bubbles. Then, each sample was loaded into clean grinding cans, with fingers gripping the mouth of the tube to avoid rapid temperature increases and degradation caused by the finger temperature. Finally, steel balls were placed inside, and the lids of the grinding cans were tightened, and then the samples were ground at 30 HZ for 30 s, and then the powder was loaded into the corresponding EP tube after grinding. Then, 500 mg (1 mL) of the powder was transferred immediately to a 20 mL headspace vial (Agilent, Palo Alto, CA, USA) containing NaCl-saturated solution to inhibit any enzyme reaction. The vials were sealed using crimp-top caps with TFE-silicone headspace septa (Agilent). At the time of SPME analysis, each vial was placed under 60 °C for 5 min, then a 120 µm DVB/CWR/PDMS fibre (Agilent) was exposed to the headspace of the sample for 15 min at 60 °C.

#### 2.5.2. GC-MS Conditions

After sampling, the desorption of the volatile compounds from the fibre coating was carried out in the injection port of the GC apparatus (Model 8890; Agilent) at 250 °C for 5 min in the splitless mode. The identification and the quantification of the volatile components were carried out using an Agilent Model 8890 GC and a 7000 D mass spectrometer (Agilent) equipped with a 30 m × 0.25 mm × 0.25 μm DB-5MS (5% phenyl–polymethylsiloxane) capillary column. Helium was used as the carrier gas at a flow rate of 1.2 mL/min. The injector temperature was kept at 250 °C, and the detector was 280 °C. The oven temperature was programmed from 40 °C (3.5 min), increasing at 10 °C/min to 100 °C, at 7 °C/min to 180 °C, at 25 °C/min to 280 °C, and held for 5 min. The mass spectra were recorded using an electron impact (EI) ionisation mode at 70 eV. The quadrupole mass detector, ion source and transfer line temperatures were set, respectively, at 150, 230, and 280 °C. For MS, the selected ion monitoring (SIM) mode was used for the identification and quantification of analysis. With the confirmed retention time, the characteristic fragment ions of the target substance were introduced into the computer method, and the qualitative and quantitative ions of the target substance were scanned at a specific fixed point to improve detection sensitivity. The retention time of all detected compounds was converted to the retention index. For each analysis target, an RI range was determined according to its reported RI in the library. The RI range was defined between (RItarget − threshold) and (RItarget + threshold), and the threshold was set as 60. All of the detected compounds within the RI range were selected as candidates for analysis.

### 2.6. Gas Chromatography Mass Spectrometry Olfactometry (GC-MS-O) Analysis

#### 2.6.1. Sample Preparation and Stir Bar Sorptive Extraction (SBSE)

During this period, the volatile compounds of the black tea samples were extracted via the stir bar sorptive extraction (SBSE) method. The black tea samples (1 g) were weighed and then brewed with boiled water (50 mL) until cooled. The tea liquid (15 mL) was filtered and poured into 20 mL headspace vials, and then NaCl (15 g) was added, and a magnetic stir bar was immersed into the tea infusion. The aroma extraction was totally conveyed within one hour under room temperature. After that, the magnetic stir bar was picked out, washed, dried, and then was transferred to a thermal desorption tube (TDU) for the subsequent analysis. Finally, the SBSE procedure was started.

#### 2.6.2. GC-MS Conditions

GC-MS was performed using the Agilent7890A/5975C (Agilent) instrument fitted with an Agilent HP-Innowax (60 m × 0.25 mm (i.d.) × 0.25 μm) capillary column. The column chamber was set to 40 °C and maintained for 5 min, after which the temperature was increased to 250 °C at a rate of 5 °C/min and maintained for 20 min. High-purity (99.999%) helium was used as the carrier gas at a flow rate of 1.8 mL/min. The samples were injected using a cold on-column technique with the temperature ranging from −30 °C to 250 °C at a rate of 15 °C/s without separation. The temperature of TDU ranged from 25 to 250 °C at a rate of 100 °C/min without separation, and the temperature of the transfer line was 260 °C. The aroma extract was split between mass spectrometry detection and olfactory detection port with a 1:1 portion. The mass spectra were recorded under the electron impact (EI) ionisation mode at 70 eV. The quadrupole mass detector, ion source, and transfer line temperatures were set, respectively, at 150 °C, 230 °C, and 250 °C. The scan width was acquired from 33 to 400 amu. Additionally, 2-nonanol was used as the internal standard.

#### 2.6.3. Olfactometry Conditions

During olfactometry detection, the aroma characteristics and intensity of the separated compounds were evaluated and described. Moreover, the intensity ranged from 1 to 4, “1” was weak, “2” was moderate, “3” was strong, and “4” was extremely strong. The descriptions of the aroma characteristics and intensity were all recorded, and compounds, especially with floral and fruity aromas, were selected for further identification.

### 2.7. Calculation of Odor Activity Value (OAV) and Aroma Character Impact (ACI)

*OAV* and *ACI* were usually applied to measure the contribution degree of the aromatic compounds. Compounds with an *OAV* higher than 1 were considered to be key aromatic compounds, and a higher *ACI* value also represented a higher contribution of relative compounds. *OAV* was calculated using the following formula:

(1) $$OAV=\frac{C_{x}}{OT_{x}}$$

where *C_x_* is the concentration of each volatile compound (μg·L^−1^); *OT_x_* is the aroma threshold of the volatile components in water (μg·L^−1^).

*ACI* was calculated using the following formula:

(2) $$ACI=\frac{O_{x}}{\sum^{\;}nOn}\times 100\%$$

where the *O_x_* is the *OAV* of each volatile compound.

### 2.8. Statistical Analysis

Difference analyses (*p* < 0.05 and *p* < 0.01 level) between JBT and FBT were performed using SPSS 18.0 software. The principal component analysis (PCA) and partial least squares discriminant analysis (PLS-DA) were both performed using SIMCA 14 software. Additionally, in the PLS-DA mode, a VIP value (Variable Importance in Projection) higher than (besides equal to) 3 was applied for the measurement.

## 3. Results

### 3.1. Sensory Evaluation Results for the Black Tea Samples

In order to have common ground for the quality description of the black tea samples, ten kinds of JBT and three kinds of FBT were sensory evaluated and described. As shown in Table 2, each JBT presented various odor characteristics such as sweet, floral, and clean aromas, especially the characteristics of a peach-like aroma with a bright orange-yellow liquid color as well as a mellow and fresh taste. In contrast, FBT tended to present a caramel-like and sweet aroma, with a red and bright liquid color as well as a mellow, thick taste.

All of the black tea samples were characterized by means of GC-MS; tea samples with a peach-like aroma were further investigated by GC-O, including JBT 2, JBT 3, JBT 6, and JBT 8 for aroma-active compounds selection. FBT 1 was used as a control.

### 3.2. Analysis of Biochemical Compositions and Color Difference of the Tea Infusions

**Figure 2:**
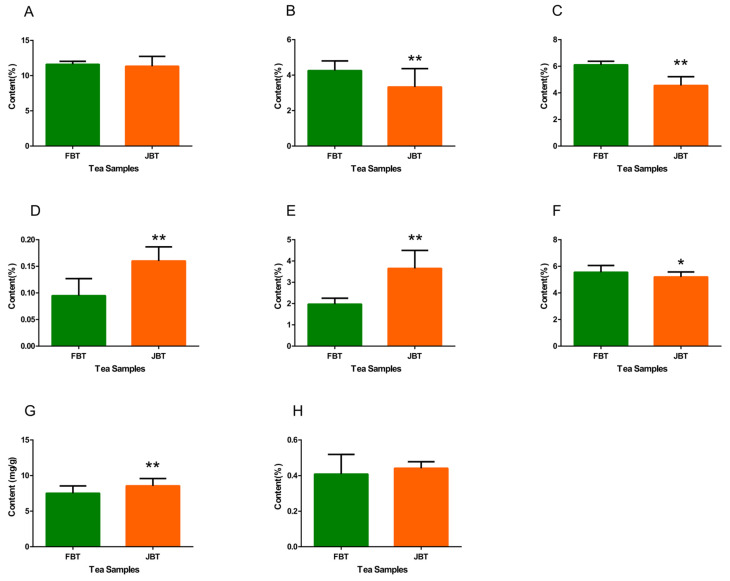
Biochemical compounds of FBT and JBT. (**A**) The content of polyphenols. (**B**) The content of free amino acids. (**C**) The content of caffeine. (**D**) The content of theaflflavins (TFs). (**E**) The content of thearubigins (TRs). (**F**) The content of theabrownins (TBs). (**G**) The content of flavonoids. (**H**) The content of water-extractable substances. Additionally, * represents a significant difference (*p* < 0.05) between FBT and JBT; ** represents an extremely significant difference (*p* < 0.01) between FBT and JBT black tea.

In total, eight kinds of biochemical compounds, including polyphenols, free amino acids, caffeine, theaflflavins (TFs), thearubigins (TRs), theabrownins (TBs), flavonoids, and water-extractable substances, were quantified using different methods based on various National Standards of the Republic of China. All of the black tea samples were clustered into two kinds depending on their processing materials. In this way, the contents of these eight compounds were compared between JBT and FBT. Figure 2 shows this comparison for each compound type (the green column represents FBT, and the orange column represents JBT). The content of free amino acids and caffeine in FBT was extremely significantly higher than those in JBT (Figure 2B,C, respectively: *p* < 0.01 in both cases). Conversely, the content of theaflflavins (TFs, Figure 2D), thearubigins (TRs, Figure 2E), and flavonoids (Figure 2G) in JBT were extremely significantly higher (*p* < 0.01 in both cases). In addition, the content of theabrownins (TBs, Figure 2F *p* < 0.05) in FBT was also significantly higher. These differences may influence the taste quality of both tea types in sensory evaluation.

An evaluation of the coloration of both tea types was performed using color indexes L*, a*, and b*, which represent lightness (L*), red–green hue (a*), and yellow–blue hue (b*), respectively. The results are shown in Table S1. The values of L*, a*, and b* for JBT infusion imply that the color of this tea infusion was close to orange–red and brighter; the values of FBT indicate that the color of this tea infusion was red and bright, which also reflects the sensory evaluation results. Additionally, a difference analysis was also conducted between JBT and FBT; capital letters represent an extremely significant difference between the samples, while lowercase letters represent a significant difference between the samples.

### 3.3. Qualitative and Quantitative Analyses of Common Aroma Components in All Black Tea Samples

The compounds were identified using GC-MS (Gas Chromatography–Mass Spectrometry) and by matching their corresponding peak in the mass spectra to values available in the NIST library. Quantitative analysis was performed using the ratio between the peak area of the substance (Figure S1) and the peak area of the internal standard (3-hexanone, 50 μg/mL).

#### 3.3.1. Aroma Profile of JBT and FBT Samples

**Figure 3:**
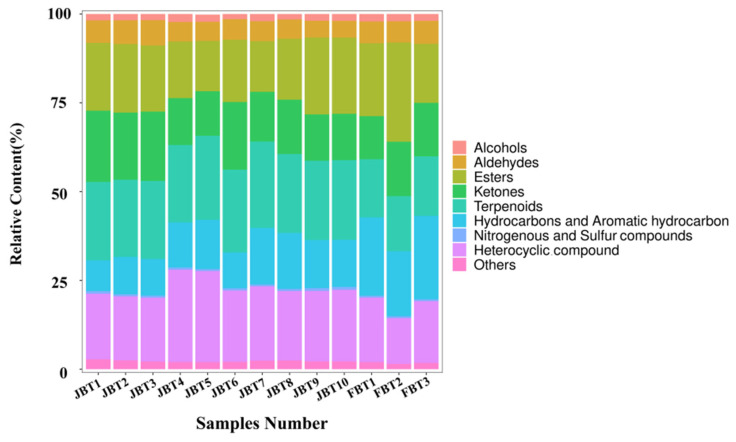
The aroma profile of JBT and FBT. Different colors correspond to the nine compound categories identified in GC-MS. JBT 1~10 indicates the sample number of Jinmudan black tea, and FBT 1~3 indicates the sample number of Fuyun black tea.

The analysis of the aroma compounds in the total black tea samples showed the presence of 949 components in total. Based on qualitative and quantitative analyses, the compounds were clustered into nine categories: alcohols, aldehydes, esters, ketones, terpenoids, hydrocarbons (including aromatic hydrocarbons), nitrogenous and sulfur compounds, heterocylic compounds, and a last category termed “others’’, in which compounds not pertaining to the previous categories were included (Figure 3). In the JBT samples, terpenoids had the highest proportion, which ranged from 21 to 23%, followed by heterocyclic compounds (17~25%) and by esters (14~21%). In the FBT samples, the esters represented the highest proportion (16~27%), followed by the hydrocarbons and aromatic hydrocarbon (18~22%) and the terpenoids (15~16%). In general, both JBT and FBT samples had a large proportion of terpenoids and esters, which tended to contribute to the floral and fruity fragrance of the processed tea.

#### 3.3.2. PCA and PLS-DA Analyses for the Potential Key Compounds (VIP)

**Figure 4:**
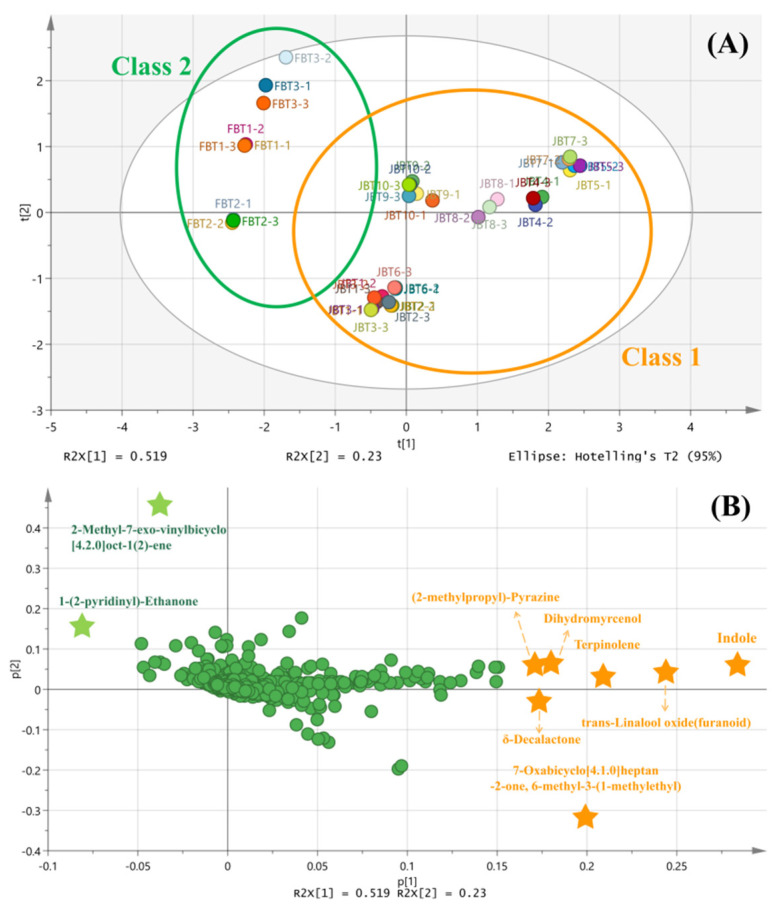
The result of the PCA analysis that was performed on JBT and FBT characteristics. (**A**) The orange circle covers the JBT 1~10, which belongs to class 1 and the green circle covers the FBT 1~3, which belongs to class 2. The contribution rate of the first principal component was 51.9%, and the second principal component was 23%. (**B**) The loading scatter plot of PCA analysis. The orange stars represent the advantageous compounds according to PC1, while the green stars represent the advantageous compounds according to PC2.

Principal Component Analysis (PCA) is a multivariate statistical method that converts multiple indicators into a few unrelated comprehensive dimensions and classifies the comprehensive indicators according to certain rules. Here, we used PCA to reveal relevant differences between JBT and FBT based on the content of compounds. As shown in Figure 4, the data could be represented according to the main components, PC1(51.9%) and PC2 (23%), which represent 74.9% of the total variance. This value allows us to describe the JBT and FBT characteristics in a reliable way. Additionally, the distance between the replicates of different samples was close, suggesting reliable repeatability. Ten kinds of JBT could be clustered into one group as class 1 (Figure 4A, orange circle), while three kinds of FBT were clustered as class 2 (Figure 4A, green circle). The various colors represent the different tea samples. This separation indicates that both tea types were highly differentiable. The loading scatter plot (Figure 4B) showed that indole, trans-linalool oxide (furanoid), terpinolene, dihydromyrcenol, (2-methylpropyl)-pyrazine, δ-decalactone, and 7-Oxabicyclo[4.1.0]heptan-2-one, 6-methyl-3-(1-methylethyl) had higher values according to PC1, which reflects the distinctive compound contents of JBT. Meanwhile, 2-Methyl-7-exo-vinylbicyclo[4.2.0]oct-1(2)-ene and 1-(2-pyridinyl)-ethanone had higher values according to PC2, which reflects that these compounds may be relevant in FBT. Thus, the PCA analysis was able to reveal the characteristic compounds of JBT and FBT.

Further investigation of PLS-DA (Partial Least-Squares Discriminant Analysis) was applied to distinguish the characteristic aroma compounds of JBT and FBT, and simultaneously, a VIP (Variable Importance Projection) score higher than (besides equal to) 3 was used as a screening criterion. Table 3 shows that in JBT, seven kinds of compounds were identified in total. Among these, indole had the highest VIP value (7.21), followed by methyl salicylate (6.45) and *δ*-decalactone (4.30), which suggests that they are key potential aromatic compounds and even contributed considerably to the peach-like aroma. Interestingly, it is noteworthy that *δ*-decalactone is also the characteristic aromatic compound of peach fruit.

Applying the same kind of analysis to FBT showed lower amounts of floral and fruity aroma compounds, which is similar to the sensory evaluation results showing that FBT has basically a caramel-like aroma. A total of six kinds of compounds had a VIP score higher than (besides equal to) 3 in FBT (Table 4). Methyl salicylate had the highest score (6.83), followed by 1-(2-pyridinyl)-ethanone(4.31), which has a nutty and popcorn-like aroma, and by 2H-pyran-2-one,tetrahydro-6-methyl(4.05), which has a burn-like aroma. Methyl salicylate thus plays an important role both in JBT and FBT, as well as in other processed teas such as white tea [28] and oolong tea [29]. This compound also contributes to the clean and fresh fragrance.

In conclusion, our analyses allowed the identification of several compounds as characteristic components of JBT and FBT aroma. It is noted that *δ*-decalactone, which confers the characteristic aroma of peach fruit [30], could be considered the potential aroma-active compound of JBT.

### 3.4. Aroma-Active Compounds (AAC) in Peach-like Black Tea Samples Identified by GC-O

GC-O (Gas Chromatography–Olfactometry) is a valuable technique for detecting and defining the flavor of the samples; it combines the separation of volatile compounds using gas chromatography with a sniffer’s olfactometry sense. Therefore, we applied this technique to characterize the aroma and intensity of four kinds of JBT samples (JBT 2, JBT 3, JBT 6, and JBT 8), which all had a significant peach-like aroma in the sensory evaluation.

**Figure 5:**
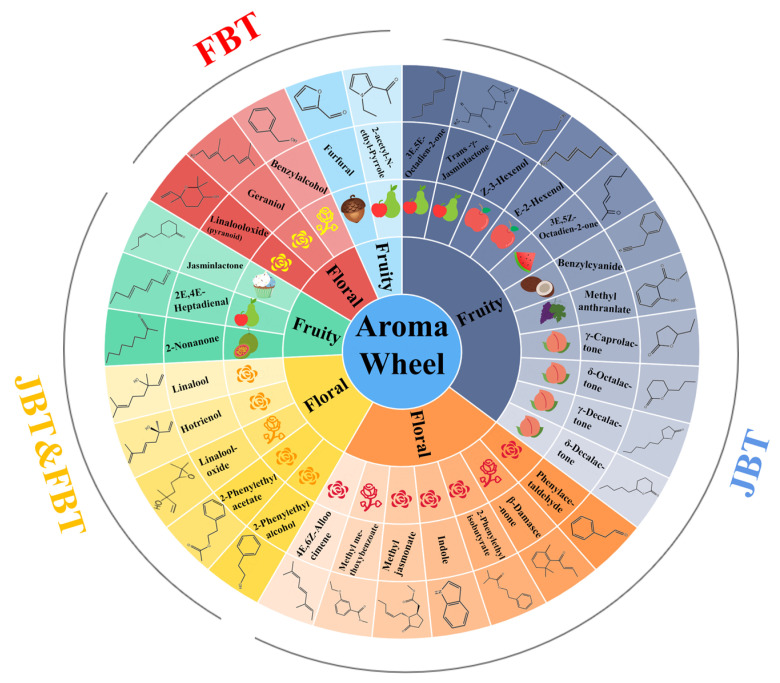
The aroma wheel of compounds in JBT and FBT. The fruit legends represent various fruity aroma characteristics of the compounds in olfactometry detection, and so as the flower legends. The capital letters and arcs divide the affiliation of the compounds.

Figure 5 shows the olfactometry results as an aroma wheel. A total of 26 kinds of aroma compounds with floral and fruity fragrances were selected as potential aroma-active compounds in JBT. Interestingly, in addition to *δ*-decalactone, other kinds of lactone, including *γ*-caprolactone, *δ*-octalactone, and *γ*-decalactone, were identified in particular samples and were related to the peach aroma [31]. However, they were not found in GC–MS. This may have been due to the fact that these four kinds of lactones were found simultaneously only in the sample of JBT 8. This result supports the previous sensory evaluation, showing that this sample had a high and significant peach-like aroma. Apart from these, jasminlactone also showed a close peach-like aroma, and it was also detected by olfactometry in all samples. Indole and coumarin shared the same retention time in olfactometry detection, and probably due to the sensory interaction between aroma compounds, these two compounds mixed and showed a strong fragrance of peach essence rather than a real peach fruit-like aroma.

In the case of FBT, the FBT 1 sample was selected for analysis due to its significant caramel-like aroma. Only 13 kinds of known aroma compounds, which contribute to the floral and fruity aromas, were distinguished. This amount was less than that in JBT; moreover, most of them were also found in JBT. As for the potential characteristic aromatic compounds of FBT, jasminlactone (creamy, fruity) and furfural (nutty) may contribute to the caramel-like aroma to some extent.

The OAVs of the potential aroma-active compounds (AAC) of the JBT were calculated by means of their Odor Thresholds (OTs). Their corresponding aroma characteristics are also described in detail in Table 5. To compare the OAV results, the mean OAVs were used. Furthermore, to compare the contribution degree of relative compounds, their ACIs were also used.

In four JBT samples, the OAVs of linalool (floral), phenylacetaldehyde (floral), and *δ*-decalactone (peach-like aroma) were higher than 1, which indicates that these compounds could be the AAC of JBT. Interestingly, in GC-MS and related analyses, *δ*-decalactone also contributed considerably to JBT characteristics. Hence, based on these results, *δ*-decalactone would play an important role in providing JBT with its characteristic peach-like aroma. Others kinds of lactone like *γ*-caprolactone played a less important role in JBT aroma composition. This may be due to their concentration and odor threshold. Moreover, the OAVs of indole, linalooloxide and methy jasmonate in particular samples and their aroma type suggest that these three compounds could be considered as the secondary AAC in JBT samples to some extent. Among these compounds, the ACI values of linaool, *δ*-decalactone, indole and hotrienol indicate that these four kinds of compounds greatly contribute to the floral and fruity (peach-like) aroma quality of JBT.

## 4. Discussion

For the purpose of describing the preliminary flavor of JBT, the sensory evaluation and biochemical compositions were conducted. As for the biochemical compositions of both two types of black tea samples, the content of free amino acids and caffeine in FBT were extremely significantly higher, while the content of theaflavins (TFs), thearubigins (TRs), and flavonoids in JBT was extremely significantly higher, which finally influenced the results of the sensory evaluation. Additionally, the index of tea infusion color was also measured. More specifically, the higher content of free amino acids and caffeine would contribute to the fresh and mellow taste of tea flavor, which is close to the sensory evaluation results of FBT; the higher content of theaflavins and thearubigins would both contribute to the taste of JBT and the bright color of the tea infusion, while the higher content of theabrownins would lead to the darker color of the FBT tea infusion.

The comparison results between the aroma quality of both two types of black tea samples show that JBT has a higher and more significant floral and fruity fragrance, especially in terms of a peach-like aroma, which did not exist in FBT. The reason may be due to the difference in processing methods, but initially, the cultivated varieties, which would be the preliminary factor [32]. The detail of this factor was also further explored by other previous studies. For example, Jinxuan and other five kinds of tea cultivars were manufactured into black tea; subsequently, the detection results showed that the tea samples made from Jinxuan possessed a higher floral aroma, which is in concert with the previous finding that this tea cultivar is also suitable for floral-aroma type black tea processing [34]. It has been found that the floral odorants in different varieties of fresh tea leaves impact the aroma quality of processed tea as well as assist the assessment of cultivar processing- suitability [35].

It is undeniable that the process methods of tea also lead to the difference in aroma quality, most in the type and the content of aroma components. Take δ-decalactone as an example, δ-decalactone has been identified as an important aromatic compound in peach fruits [36], milk products [37,38], and some specific tea samples. However, this compound has received less attention than other volatile compounds, such as genaniol or linalool. In our study, we surprisingly found that *δ*-decalactone was the AAC of innovative JBT, and we suspected that the differences between tea processing methods should be taken into account. For example, previous studies showed that *δ*-decalactone was also determined in Wuyi Rock tea, but due to the baking method, this kind of volatile was not approved as AAC [33]. Furthermore, a previous study found that *δ*-decalactone and jasmine lactone were both identified in traditional JBT, but just jasmine lactone was characterized as AAC that contributes to the fruity aroma [39]. Contrarily, in our study, *δ*-decalactone was characterized as AAC of peach-like JBT while jasmine lactone was not. It could be speculated that different processing methods led to this result. In our research, we selected the innovative JBT as samples, which integrate the turning-over method during production and consequently have a more significant peach-like aroma.

A previous study found that fatty acids and its derivative are the important precursors supplied for fruity aroma volatile, which belong to straight chain aliphatic alcohols, aldehydes, ketones, and esters [40]. Apart from these, it has also been found that turning-over could facilitate the formation and accumulation of aroma volatile, especially the fatty acids, which contribute to fruity aroma during the processing period of oolong tea [41,42]. Therefore, the processing methods play an indispensable role in the formation of tea aroma quality. The other key component identified in this study, linalool, which shows a pleasant floral fragrance in tea, has also been found to show dynamic change during processing [43]. In more detail, the level and enantiomeric distributions of linalool in various kinds of tea cultivars were detected, and the study found that processing methods also had a large impact on the linalool level based on the results that R-(−)-Linalool and S-(+)-linalool reached maximum level during the rolling period of black tea while the level declining drastically during green tea processing [44]. Apart from these, indole plays an important role in Jinmudan aroma quality, and it is a signal substance that can prime defenses and resistance of plants [45]. This component was found to accumulate during the turning-over process, and finally, the formation mechanism was revealed [46].

## 5. Conclusions

In this study, the basic sensory evaluation and biochemical compositions of innovative Jinmudan black tea were conveyed, and then the aroma profile of innovative Jinmudan black tea was revealed, which possessed a floral and fruity aroma, especially a peach-like aroma. As a result, based on GC-O detection combined with OAV and ACI analysis, the key aroma-active components including linalool, phenylacetaldehyde, and δ-decalactone were selected, which were associated with a floral and fruity aroma. Additionally, indole, linalooloxide, and methy jasmonate could be considered to be secondary aroma-active compounds. It can be suggested that δ-decalactone may contribute to the peach-like aroma to some extent in innovative Jinmudan black tea, while other components may contribute to the floral aroma. According to the comparison between Jinmudan and Fuyun black tea, we could conclude that the peach-like aroma of Jinmudan black tea mostly originated from its cultivated variety and processing method, from which not a single one can be omitted. In the near future, further investigation is warranted that includes the identification of the aroma precursors in fresh tea leaves and the dynamic changes of the key aroma compounds during the stage of the tea manufacturing process.

## Figures and Tables

**Table 1:** The subject and National Standards of the Republic of China have been applied in the biochemical compounds quantification.

Subject	National Standard of China
Polyphenols	GB/T 8313 2002
Free amino acids	GB/T 8314 2002
Caffeine	GB/T 8312 2013
Theaflavins (TFs), Thearubigins (TRs), and Theabrownins (TBs)	Zhang [27]
Flavonoids	GB/T 8313 2002
Water-extractable substances	GB/T 8305 2013

**Table 2:** Sensory quality of JBT and FBT samples.

Tea Samples	Sensory Quality		
	Liquor Color	Aroma	Taste
JBT 1	Orange with yellow and bright	High and pure	Smooth
JBT 2	Orange with yellow and bright	High and long-lasting peach-like aroma	Smooth and mellow
JBT 3	Orange with yellow and bright	Pure with peach-like aroma	mellow
JBT 4	Orange with yellow and bright	Sweet and pure but with aging leaf odour	Mellow and fresh, after-taste
JBT 5	Orange with red and bright	High and pure but with aging leaf odour	Thick and mellow with aging leaf odour
JBT 6	Orange with red and bright	High and long-lasting with peach-like aroma	Mellow and fresh
JBT 7	Orange with red and bright	Aging laf odour	Mellow but with aging leaf odour
JBT 8	Orange with red and bright	High and significant peach-like aroma	Astringent and green
JBT 9	Orange with red and bright	High and clean	Thick and fresh
JBT 10	Orange with red and bright	High and long-lasting	Thick and fresh
FBT 1	Red and bright	Significant caramel-like aroma	Mellow
FBT 2	Red and bright with a golden circle	Caramel-like with a floral aroma	Fresh and thick
FBT 3	Red and bright	Caramel-like and pure	Mellow and fresh

**Table 3:** VIP higher than (besides equal to) 3 compounds in JBT.

Compounds	CAS	Class	VIP	Aroma Type
Indole	120-72-9	Heterocyclic Compound	7.21	Floral
Methyl salicylate	119-36-8	Ester	6.45	Green, holly oil-like
*δ*-Decalactone	705-86-2	Ester	4.30	Peach-like, creamy
trans-beta-Ocimene	3779-61-1	Terpenoids	3.58	Floral, green
(E)-4,8-Dimethylnona-1,3,7-triene	19945-61-0	Terpenoids	3.46	Floral
cis-alpha-Bisabolene	29837-07-8	Terpenoids	3.17	Green
Hexanoic acid, hexyl ester	6378-65-0	Ester	3.00	Fruity

**Table 4:** VIP higher than (besides equal to) 3 compounds in FBT.

Compounds	CAS	Class	VIP	Aroma Type
Methyl salicylate	119-36-8	Ester	6.83	Green, holly oil-like
1-(2-pyridinyl)-Ethanone	1122-62-9	Heterocyclic compound	4.31	Nutty, popcorn-like
2H-Pyran-2-one, tetrahydro-6-methyl-	823-22-3	Heterocyclic compound	4.05	Fruity, burn-like
1-ethyl-3-methyl-Benzene	620-14-4	Aromatics	3.71	NF
2H-Pyran-3-ol, 6-ethenyltetrahydro-2,2,6-trimethyl	14049-11-7	Heterocyclic compound	3.61	NF
trans-Linalool oxide (furanoid)	34995-77-2	Heterocyclic compound	3.60	Floral

**Table 5:** The aroma character, OT value, OAV, and ACI values of related compounds in JBT.

Compounds	Aroma Character	Aroma Intensity				OT (µg/L)	OAV				ACI (%)			
		JBT2	JBT3	JBT6	JBT8		JBT2	JBT3	JBT6	JBT8	JBT2	JBT3	JBT6	JBT8
Alloocimene 4E,6Z-	Orange, floral	3	3	2	2	NF	-	-	-	-	-	-	-	-
Hexenol, 3Z-	Green apple-like	4	3	2	4	70 ^a^	0.123	0.167	0.121	0.221	0.46	1.47	0.53	1.07
Nonanone, 2-	Passion fruit-like	4	2	4	3	200 ^a^	0.077	0.159	0.172	0.158	0.29	1.39	0.76	0.76
Hexenol, 2E-	Green apple-like	-	3	-	3	100 [32]	-	0.013	-	0.005	-	0.12	-	0.25
Linalooloxide	Rose-like	4	-	2	2	190 [19]	0.163	-	0.223	2.409	0.62	0.30	0.98	11.67
Heptadienal, 2E,4E-	Fruity	4	4	2	3	NF	-	-	-	-	-	-	-	-
Octadien-2-one, 3E,5Z-	Watermelon-like	3	4	3	3	NF	-	-	-	-	-	-	-	-
Linalool	Floral, orange-like	4	3	3	3	6 ^a^	15.595	4.348	14.011	8.833	59.13	38.29	61.5	42.75
Octadien-2-one, 3E,5E-	Fruity	3	3	3	3	NF	-	-	-	-	-	-	-	-
Hotrienol	Floral	3	4	2	3	110 [32]	0.755	0.003	0.805	0.782	2.86	0.03	3.54	3.79
Phenylacetaldehyde	Floral, hyacinth-like	4	3	4	3	4 ^a^	1.859	3.912	2.066	1.344	0.29	34.45	0.08	6.51
γ-Caprolactone	Peach-like	3	3	3	3	50 [19]	0.071	0.031	0.114	0.101	0.27	0.27	0.50	0.49
β-Damascenone	Rose-like	-	-	-	3	NF	-	-	-	-	-	-	-	-
2-Phenylethyl isobutyrate	Floral, rose-like	-	2	2	3	NF	-	-	-	-	-	-	-	-
2-Phenylethyl alcohol	Floral, fruity	2	-	1	2	390 [19]	0.365	-	0.238	0.195	1.38	-	1.05	0.94
Benzyl cyanide	Coconut-like, woody	3	3	-	4	NF	-	-	-	-	-	-	-	-
δ-Octalactone	Peach-like, creamy	3	2	3	4	400 ^a^	0.008	0.006	0.002	0.001	0.03	0.05	0.01	0.49
Methyl methoxybenzoate	Rose-like	-	-	-	3	NF	-	-	-	-	-	-	-	-
γ-Decalactone	Peach-like	-	-	-	3	11 ^a^	-	-	-	0.062	-	-	-	0.29
Trans-γ-Jasminlactone	fruity	-	3	-	2	NF	-	-	-	-	-	-	-	-
δ-Decalactone	Peach-like, osmanthus flower-like	3	2	2	4	100 ^a^	1.476	1.448	1.094	1.499	5.59	12.72	4.80	7.25
Methyl anthranlate	Grape juice-like	3	1	2	3	3 [32]	0.061	0.353	0.206	0.626	0.23	3.11	0.90	3.03
Jasminlactone	Creamy, closely peach-like	3	2	3	4	2000 ^a^	0.001	0.265	0.802	0.004	0.002	2.34	3.52	0.02
Methyl jasmonate	Floral	4	2	2	2	3 [19]	4.28	0.047	0.629	3.569	16.23	0.41	2.76	17.28
Indole	Floral	3	4	3	4	140 ^a^	1.215	0.337	1.291	0.586	4.61	2.96	5.67	2.83
2-Phenylethyl acetate	Floral	3	2	3	2	249 [33]	0.076	0.023	0.014	0.063	0.288	0.21	0.06	0.31

‘NF’ means data were not found in the literature; ‘-’ means compounds were not caught and distinguished; Odor Thresholds(OTs) accessed from; ‘^a^’ The LRI and Odor Database and related reference.

## Data Availability

Data is contained within the article or supplementary material.

## Supplementary Materials

The following supporting information can be downloaded at: https://www.mdpi.com/article/10.3390/foods12091794/s1, Figure S1: The total iron current of the samples; Table S1: Color difference of the JBT and FBT infusions.
