# Supplementary material for: Identification of Key Components Responsible for the Aromatic Quality of Jinmudan Black Tea by Means of Molecular Sensory Science
Source: Foods. 2023 Apr 26;12(9):1794. doi: 10.3390/foods12091794 (PMC10178690; doi:10.3390/foods12091794)
Supplement: Supplementary file 1 [file foods-12-01794-s001.zip › foods-2326223-supplementary.pdf]

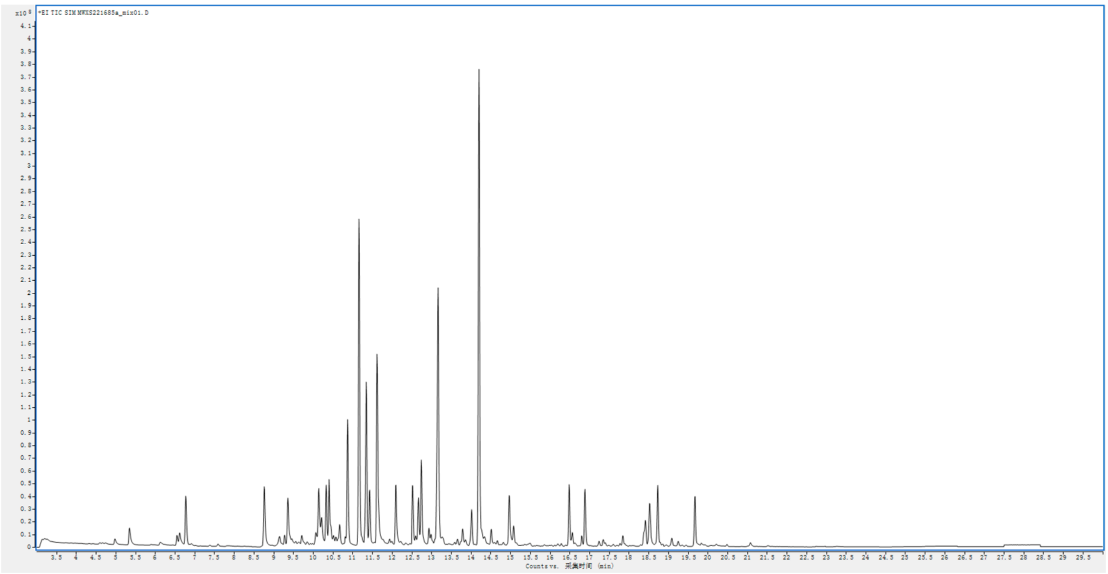

Figure S1. The total iron current of the samples. The horizontal ordinate represents the retention time(Rt) of the volatile components, the vertical axis represents the ion current intensity for ion detection(cps, count per second).

Table S1. Color difference of the JBT and FBT infusions.

	JBT1	JBT2	JBT3	JBT4	JBT5	JBT6	JBT7	JBT8	JBT9	JBT10	FBT1	FBT2	FBT3
L^{*}	27.25±0.03^{Bb}	26.96±0.01^{CcDd}	27.19±0.01^{Bb}	27.81±0.01^{Aa}	27.15±0.01^{BCbc}	26.47±0.2^{Dd}	25.92±0.05^{Ff}	25.5±0.02^{Gg}	25.67±0.01^{Gg}	27.94±0.01^{Aa}	26.94±0.01^{Dd}	26.98±0.02^{CcDd}	26.80±0.02^{Cc}
a^{*}	2.38±0.01^{Aa}	2.28±0.02^{Aa}	2.26±0.02^{Aa}	2.61±0.3^{Aa}	2.39±0.02^{Aa}	2.42±0.10^{Aa}	2.35±0.08^{Aa}	2.37±0.03^{Aa}	2.35±0.01^{Aa}	2.44±0.01^{Aa}	2.19±0.01^{Aa}	2.33±0.06^{Aa}	2.19±0.01^{Aa}
b^{*}	-0.18±0.02^{Ee}	-0.63±0.2^{Ii}	-0.67±0.02^{Ii}	0.55±0.01^{Bb}	-0.05±0.02^{Dd}	-0.17±0.02^{Ee}	0.63±0.01^{Aa}	-0.3±0.02^{Ff}	0.05±0.01^{Cc}	-0.92±0.02^{Kk}	-0.53±0.02^{Hh}	-0.37±0.01^{Gg}	-0.75±0.02^{Ji}

The capital letters represents extremely significant difference between samples and the lowercase letters represents significant difference between samples.
